# Estrous Cycle Impacts Fear Extinction and Relapse in Female Adolescent Rats

**DOI:** 10.1002/dev.70084

**Published:** 2025-09-09

**Authors:** August Gable, Rick Richardson, Kathryn D. Baker

**Affiliations:** ^1^ School of Psychology UNSW Sydney Sydney New South Wales Australia; ^2^ Department of Psychology, Counselling and Therapy, School of Psychology & Public Health La Trobe University Melbourne Victoria Australia

**Keywords:** adolescence, estradiol, extinction, female, renewal

## Abstract

Adolescent male rodents and humans exhibit impairments in extinguishing learned fear. Here, we investigated whether female adolescent rats exhibit such impairments and if extinction is affected by the estrous cycle as in adults. Following fear conditioning to a discrete cue, female adolescent Sprague Dawley rats were extinguished either around the onset of puberty, when estrous cycling begins, or across different stages of the estrous cycle. Both extinction retention and renewal (a form of relapse) were assessed. Peri‐pubertal females had comparable freezing during extinction training and tests of extinction retention and fear renewal as age‐matched males. They were noted to generally be in metestrus, a low estradiol phase, at extinction training. Postpubertal females that received extinction training in proestrus (high estradiol phase), but not metestrus (low estradiol phase), had lower freezing during extinction training and retention than males; males exhibited more freezing during a renewal test than both groups of females. Our findings suggest that female adolescent rats have reduced fear during extinction training and retention compared to males only when extinguished in a high‐estradiol phase. These findings suggest fear inhibition fluctuates across the estrous cycle in adolescence, and estradiol may protect females against impairments in fear extinction during this developmental period.

## 1 Introduction

Women are more likely to experience anxiety, with more disabling symptoms, than men (Kessler et al. 2005; McEvoy et al. 2011; McLean et al. 2011). This sex difference may emerge in adolescence, as higher rates of anxiety and post‐traumatic stress disorder are often found in teenage girls than in boys (Kessler et al. 2012; McEvoy et al. 2011; Merikangas et al. 2010), but the same sexual dimorphism is not observed in children (Copeland et al. 2014; Ford et al. 2003). Puberty appears to be a tipping point for the emergence of sex differences in anxiety (Hayward and Sanborn 2002; Patton et al. 1996), as both anxiety and panic attacks are positively associated with pubertal development in girls (Carter et al. 2011; Deardorff et al. 2007; Hayward and Sanborn 2002). Related work indicates that pubertal maturation is associated with increased fear responses to threat cues in girls and boys (Stenson et al. 2021).

Anxiety disorders are commonly treated with exposure‐based therapy, which involves procedures based on the extinction of learned fear (Hamlett et al. 2023; McNally 2007). Unfortunately, not all individuals who undergo exposure‐based therapy show improvements (DiMauro et al. 2013), and relapse is common in those individuals who do show therapeutic gains (Brown and Barlow 1995; Gloster et al. 2013). It is also concerning that remission rates have been reported to be lower in females than males during adolescence and early adulthood (Ginsburg et al. 2018, 2014), suggesting that residual anxiety symptoms are more likely to occur in females. A deeper understanding of extinction in females may help improve behavioral approaches to reducing anxiety in teenage girls.

Female‐unique factors are known to modulate extinction. For example, adult female rats and women have a differential response to fear extinction across the various stages of their reproductive cycles (i.e., the estrous cycle in rodents and the menstrual cycle in women) (Graham and Scott 2018a; Milad et al. 2009, 2010; Milligan‐Saville and Graham 2016; Pineles et al. 2016). Extinction is impaired in females during periods of low ovarian hormones (i.e., estradiol and progesterone) and facilitated when ovarian hormones are high (Glover et al. 2012; Graham and Scott 2018a; S. Li and Graham 2016; Milad et al. 2009, 2010; Rey et al. 2014; Wegerer et al. 2014). More specifically, female adult rats receiving extinction training in the proestrus phase of the estrous cycle (when estradiol levels are high) have lower levels of freezing at an extinction retention test the next day relative to those receiving extinction training in the metestrus phase (when estradiol levels are low) (Graham and Milad 2013; Graham and Scott 2018a; Milad et al. 2009; Milligan‐Saville and Graham 2016; Rey et al. 2014). Similarly, relative to women at the end of the luteal (low‐hormone) phase, naturally cycling women in the follicular (high‐hormone) phase of the menstrual cycle have better extinction memory during recall (i.e., retention) testing (S. Li and Graham 2016; Milad et al. 2010). There is one notable exception in that cyclic ovarian hormones do not modulate fear extinction after pregnancy in either adult female rats or women (Milligan‐Saville and Graham 2016; Pestana et al. 2021; Tang and Graham 2020).

There is also evidence that there are notable differences in extinction across developmental stages, such that adolescents, both rodent and human, have impaired learning and/or retention of extinction relative to adults and juveniles (Baker and Richardson 2015; Bisby et al. 2021; Ganella et al. 2017; Hefner and Holmes 2007; Kim et al. 2011; McCallum et al. 2010; Pattwell et al. 2012; but see Britton et al. 2013). This deficit in adolescents is associated with functional differences in the recruitment of prefrontal‐amygdala networks necessary for extinction in adults (Baker and Richardson 2015; Kim et al. 2011; Zimmermann et al. 2023). However, most of the preclinical studies of fear extinction in developing animals have been conducted in males (see Bisby et al. 2021 for a systematic review of the relevant research in adolescence), mirroring the sex bias in most preclinical research in learning, memory, and neuroscience (Beery 2018; Lebron‐Milad and Milad 2012) and on fear and anxiety even as recently as 2021 (Kaluve et al. 2022).

The sex bias in rodent studies is hindering the advancement of our understanding of whether fear extinction in adolescence differs in males and females. In the few studies that have been done to date, adolescent male rats typically exhibit impaired extinction retention relative to older and younger ages, despite similar acquisition and initial extinction of conditioned fear (for review, see Bisby et al. 2021). In contrast, female adolescents were found to learn and retain fear extinction at long‐term intervals similarly to adults (McCormick et al. 2013). One study found similar effects in mice, reporting that male, but not female, adolescent (Postnatal [P] Day 29) mice had more recovery of extinguished fear across extinction sessions than adults (Glavonic et al. 2023). However, in contrast to these reported sex differences, another study in adolescent (P35) male and female rats reported similar extinction learning and retention (Chocyk et al. 2014). Notably, none of the above studies examined the potential influence of puberty or fluctuations of the estrous cycle. Only one study to date has examined the effect of the estrous cycle on fear extinction in adolescent female rodents. This study reported that adolescent female rats (P35) in low estradiol phases (metestrus or diestrus) exhibited impaired extinction learning and retention relative to males, but similar‐aged females that were prepubertal (and therefore had not commenced estrous cycling) did not have any impairments (Perry et al. 2020). More research on the effects of puberty and the estrous cycle in adolescent females is needed.

In addition to the dearth of studies examining the extinction of learned fear in female adolescent rodents, another significant gap is the lack of studies on the relapse of extinguished fear, such as renewal. Conditioned responses often renew in a different physical context from the extinction context (Bouton and Bolles 1979). One study in males reported equivalent renewal when testing occurred in the original conditioning context (i.e., ABA renewal) in juvenile, adolescent, and adult rats (at P24, P35, and P70, respectively) (Kim et al. 2011). However, adult females have been reported to exhibit less renewal of extinguished conditioned responses than males in aversive (Binette et al. 2022) and appetitive conditioning tasks (Anderson and Petrovich 2015). When this attenuated relapse in females emerges is not yet clear, but it could appear in adolescence, given that juvenile (P25) and infant (P18) female rats exhibit fear renewal (Park et al. 2020). In that work, adolescents were not tested, nor were males. Determining if there are sex differences in renewal among juveniles and adolescents, and if age (or puberty) affects renewal in females, would provide valuable insights into fear relapse in developing females.

An important factor to consider when examining extinction in female rats during adolescence is that puberty occurs within this developmental stage. At the onset of puberty, circulating ovarian hormones (estradiol and progesterone) increase with the first ovulation (Advis et al. 1979; Döhler and Wuttke 1974; Evans 1986; Ojeda et al. 1980; Parker and Mahesh 1976). Circulating estradiol levels double and progesterone levels surge fivefold at puberty relative to prepubertal levels (Parker and Mahesh 1976). Puberty onset can be detected in rats by vaginal opening, which occurs around P35–38 (Döhler and Wuttke 1974). In rats, the first ovulation is coupled with vaginal opening and the commencement of estrous cycling (Becker et al. 2005; Lynch 2008). Although vaginal opening and puberty coincide with increased estradiol and progesterone levels, there is conflicting evidence about when estradiol levels subside during the first estrous cycle, with some reporting that they remain elevated until at least the day after vaginal opening (Parker and Mahesh 1976), whereas others have reported that they decrease the day after vaginal opening (Advis et al. 1979; Ojeda et al. 1980), like progesterone (Parker and Mahesh 1976). Thus, ovarian hormone levels surge and wane in peri‐pubertal female rats, and this fluctuation may influence the effectiveness of extinction.

In this study, we aimed to replicate past findings of extinction deficits in adolescent females as in males but extend prior work by including an explicit comparison to juvenile males and females and to determine if there are sex and estrous cycle differences in renewal of extinguished fear in adolescence. Adolescent female rats received extinction training either around the onset of puberty (Experiment 1) or across different stages of the estrous cycle (Experiment 2). To distinguish the adolescent cohorts, we refer to females in Experiment 1 as peri‐pubertal and those in Experiment 2 as mid‐adolescents based on their age at extinction training. Same‐age males were included in each experiment for comparison. Also, given the conflicting evidence about circulating estradiol levels around the time of puberty onset in female rodents, we examined serum estradiol levels at similar ages in untrained animals (Experiment 3). It was predicted that, relative to juveniles, female adolescent rats would have impaired extinction retention like male adolescents. We explored whether extinction retention was improved when extinction training occurred in proestrus, a high‐estradiol phase of the estrous cycle, as has been reported in adults (Graham and Milad 2013; Graham and Scott 2018a; Milad et al. 2009; Milligan‐Saville and Graham 2016; Rey et al. 2014), or is unaffected, as previously reported in adolescent rats (Perry et al. 2020). In addition, studies in humans report that earlier pubertal maturation appears to be associated with increased fear response to threat cues in girls and boys (Stenson et al. 2021). Given that we measured puberty onset in females, we were able to explore whether earlier pubertal timing is associated with poorer long‐term response to extinction (Experiment 2). We predicted that higher fear after extinction (i.e., at extinction retention and renewal tests) would be associated with earlier pubertal onset in females. In past work, we did not find that male adolescent rats differed on extinction learning or retention before or after puberty, but older adolescents had a faster loss of fear during an extinction retention test than younger adolescents (Wall et al. 2023). Therefore, we predicted that higher fear after extinction (i.e., at extinction retention and renewal tests) would be associated with younger age within adolescence in both males and females.

## 2 Materials and Method

### 2.1 Animals

Experimentally naive male and female Sprague Dawley rats (*N* = 219) were obtained from the UNSW Sydney (UNSW) School of Psychology breeding colony. The animals were weaned at P20–22 and housed in groups of four with same‐sex animals in conditions similar to those in our previous studies (Baker et al. 2013; Baker and Richardson 2015). Block randomization was used to allocate rats to treatment conditions. No more than one rat per litter was assigned to any experimental group (Cowan and Richardson 2018). All animals were treated following the *Australian Code of Practice for the Care and Use of Animals for Scientific Purposes* (8th edition, 2013), and all procedures were approved by the Animal Care and Ethics Committee at UNSW.

### 2.2 Fear Conditioning and Extinction Procedures

**FIGURE 1:**
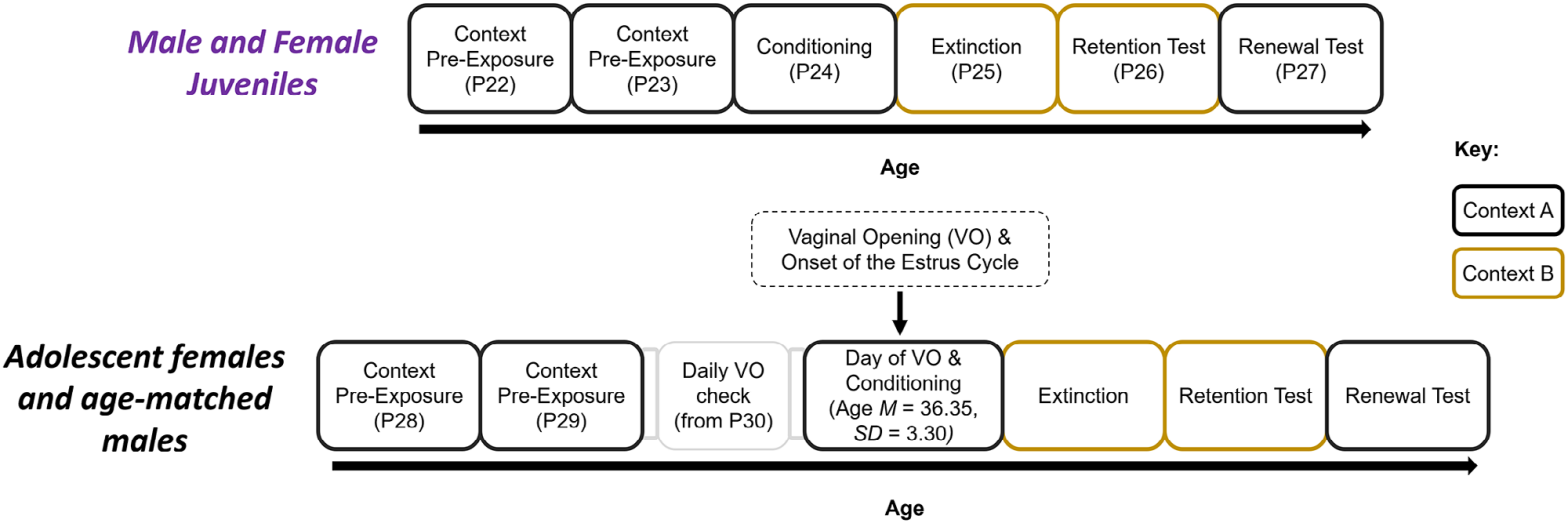
Schedule for behavioral procedures for Experiment 1. Adolescents were on average around 36–37 days old at conditioning (females: *M* = 36.35, SD = 3.30; males: *M* = 37.82, SD = 3.59). Conditioning, extinction training, and both tests occurred on consecutive days.

Behavioral procedures and apparatus were similar to those used in our previous studies (Baker et al. 2013; Baker and Richardson 2015). In brief, handling, conditioning, extinction training, the extinction retention test, and the renewal test occurred around 24 h apart in Experiment 1 (see Figure 1). The schedule of behavioral procedures was slightly modified in Experiment 2 so that extinction training was timed to the estrous cycle stage in the female rats (see Figure 4). Two contexts (A and B), which differed in visual and tactile features, were used for different stages of the behavioral procedures; conditioning and the renewal test occurred in Context A, whereas extinction training and the extinction retention test occurred in Context B. Animals were handled for 4 min each day on two consecutive days. After each bout of handling, the animals were preexposed to Context A for 8 min. During conditioning, the CS and US were paired three times with intertrial intervals of 135 and 85 s. The CS was a 10 s white noise that co‐terminated with a 1 s footshock unconditioned stimulus (US; 0.45 mA) delivered through a grid floor. During extinction training, there were 30 presentations of the CS (each 10 s), each separated by a 10 s intertrial interval. The extinction retention test and the renewal test identically consisted of a single 2‐min presentation of the CS, with extinction retention being tested in Context B and renewal tested in Context A. A 2‐min baseline period was included in each behavioral session.

### 2.3 Puberty and Estrous Cycle Phase Determination

The onset of puberty in females was determined by vaginal opening (Döhler and Wuttke 1974; Evans 1986). Each female rat, from P29 or P30, was assessed daily for vaginal opening between 7:00 a.m. and 9:00 a.m. Once opened, the estrous cycle was determined by examination of cells collected by a vaginal lavage (via the pipette method) following procedures established by Becker et al. (2005). Lavage fluid was dispensed onto microscope slides, air‐dried, stained with Quick Dip Stain Kit (POCD Scientific), and assessed by light microscopy for various cell types, indicating the phase of the estrous cycle. Proestrus was identified by the presence of nucleated epithelial cells, and estrus was determined by the predominance of cornified and anucleated cells. The presence of predominantly leukocytes identified metestrus, whereas the presence of predominantly leukocytes and mucous identified diestrus (Becker et al. 2005).

### 2.4 Serological Estradiol Analysis

In Experiment 3, the estrous cycle of behaviorally naive adolescent females was monitored via vaginal cytology, and animals were euthanized on different days of the first or second estrous cycle to quantify serum estradiol levels matching the timing of behavioral procedures in earlier experiments. Estradiol levels were compared across the day of and the day after vaginal opening, as well as the day of proestrus and metestrus during the second estrous cycle. Up to four animals were kept in each cage, and trunk blood was only collected from socially housed animals (i.e., no animals were singly housed following a cage mate being euthanized). Trunk blood was collected from animals immediately after euthanasia with carbon dioxide and allowed to coagulate for 30 min. The serum was separated by centrifugation at 3000 Rcf for 15 min at 4°C, transferred to clean microtubes, and stored at −30°C. Serum estradiol concentrations were determined using a commercially available enzyme‐linked immunosorbent assay kit (ab108667, Abcam, Australia) and a microplate reader (Bio‐Rad, Australia) following the manufacturer's protocol. Samples were run in duplicate.

### 2.5 Analysis

Freezing behavior was the dependent variable for learned fear and was defined as the absence of all movement except that which is needed for respiration (Fanselow 1980). Each rat was scored every 3 s as either freezing or not freezing during baseline and all CS presentations. A percentage score was calculated for each rat to determine the proportion of total freezing observations during baseline or CS presentations. Six extinction trials were averaged to represent an extinction block for statistical analyses of within‐session extinction. A second observer who was blind to experimental conditions scored ∼30% of freezing data at the retention and renewal tests for each experiment. Inter‐rater reliability was very high for both baseline, *r* = 0.94–0.96, and CS‐elicited freezing, *r* = 0.93–0.97. Estradiol levels were expressed as a percentage of the mean data from metestrus controls on each plate.

Statistical analyses were conducted using SPSS version 29, and GraphPad Prism was used for figures. Levels of CS‐elicited freezing during fear conditioning or extinction training were analyzed using a mixed‐design ANOVA, with age and sex (Experiment 1) or group (Experiment 2) as between‐subjects factors and conditioning trial or extinction block as a within‐subjects (repeated measures) factor. If the assumption of sphericity was violated for any mixed‐design ANOVA, the Greenhouse–Geisser procedure was used, and the adjusted degrees of freedom were reported. CS‐elicited freezing in the retention and renewal tests was analyzed using ANOVA with age and sex (Experiment 1) or group (Experiment 2) as between‐subject factors. In those analyses, given only three groups were being compared, Student–Newman–Keuls (SNK) post hoc tests were used for follow‐up contrasts (Proschan and Brittain 2020; Shaffer 2007). Independent samples *t*‐tests or one‐way ANOVA were used to compare the mean age at extinction training or vaginal opening across groups. Serum estradiol levels were analyzed using one‐way between‐subjects ANOVA with Tukey's HSD post hoc tests (as four groups were being compared). Effect sizes were reported as partial eta squared (*η_p_* ^2^) for ANOVA or Cohen's *d* for *t*‐tests following Richardson (2011) and Cohen's (1988) categorization of small (*η_p_* ^2^ = 0.01; *d* = 0.2), medium (*η_p_* ^2^ = 0.06; *d* = 0.5), and large (*η_p_* ^2^ = 0.14; *d* = 0.8) effect sizes. In Experiment 2, where the age of extinction varied, Pearson's correlation analyses were used to separately correlate the age of puberty (i.e., vaginal opening; for females only) and age at extinction training with CS‐elicited freezing during the retention and renewal tests in each group. An alpha value of *p *= 0.05 was applied to all analyses.

## 3 Results

### 3.1 Experiment 1

Experiment 1 examined the effect of pubertal onset on the retention of fear extinction in peri‐pubertal female adolescent rats using a factorial design with age (juvenile or adolescent) and sex (male or female) as factors. The behavioral design for the experiment is shown in Figure 1. At P22, the juveniles were given two sequential days of handling and pre‐exposure, followed by fear conditioning the next day (at P24). Then, sequentially for the following 3 days, the juvenile male and female animals were given extinction training, an extinction retention test, and a renewal test, respectively. Rats in the female adolescent group were given two sequential days of handling and pre‐exposure at P28 and P29. From P30 onwards, they were assessed daily for the onset of puberty, as indicated by vaginal opening. On that day, female rats were given fear conditioning; on subsequent days, they received extinction training, an extinction retention test, and a renewal test (see Figure 1). All handling and behavioral procedures for male adolescents were age‐matched to those of the female adolescents. In addition, male adolescents were handled daily in a similar manner as female adolescents were handled during lavage procedures. All handling and procedures occurred between P22 and P27 for juveniles or between P28 and P50 for adolescents—ages within the window of juvenility and adolescence, respectively, in rats (Schneider 2013).

#### 3.1.1 Exclusions

Out of 85 animals, 7 animals (2 male juveniles, 3 female juveniles, and 2 male adolescents) were excluded from the behavioral analyses for having levels of CS‐elicited freezing on average less than 15% across Extinction Blocks 1 and 2, indicating there was poor initial acquisition of conditioned fear. Four animals were deemed statistical outliers and excluded for being more than 2 standard deviations away from the mean during either the extinction retention or renewal test (one male adolescent, one female juvenile, and two male juveniles). In Experiment 1, the renewal test procedure was added after some initial data had been collected; the first rats in three of the conditions (i.e., three female adolescents, four male adolescents, and four male juveniles) received conditioning, extinction training, and the extinction retention test but not renewal testing, resulting in somewhat smaller group sizes for that test for these groups. The final group sizes for Experiment 1 at conditioning, extinction training, and the extinction retention test were *n* = 18 for the male juvenile group (*n* = 14 at renewal), *n* = 19 for the female juvenile group (*n* = 19 at renewal), *n* = 17 for the male adolescent group (*n* = 13 at renewal), and *n* = 20 for the female adolescent group (*n* = 17 at renewal).

#### 3.1.2 Vaginal Cytology

**FIGURE 2:**
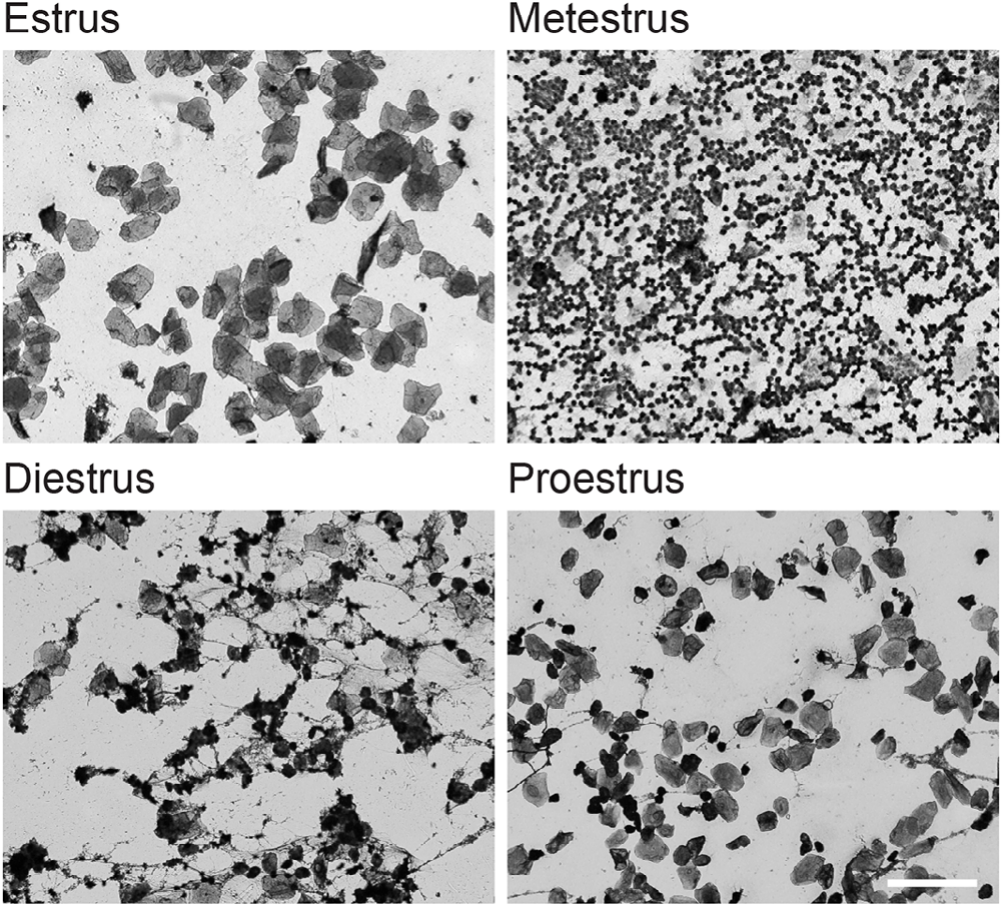
Representative images of cells taken via vaginal lavage in Experiment 1. Estrus was indicated by the predominance of cornified cells and the absence of leukocytes. Metestrus was identified by high cellularity of leukocytes. Diestrus was indicated by a mix of mucous, leukocytes, anucleated, and nucleated cells. Proestrus was identified by a predominance of nucleated cells, some cornified cells, and the absence of leukocytes. Scale bar 100 µm.

**FIGURE 3:**
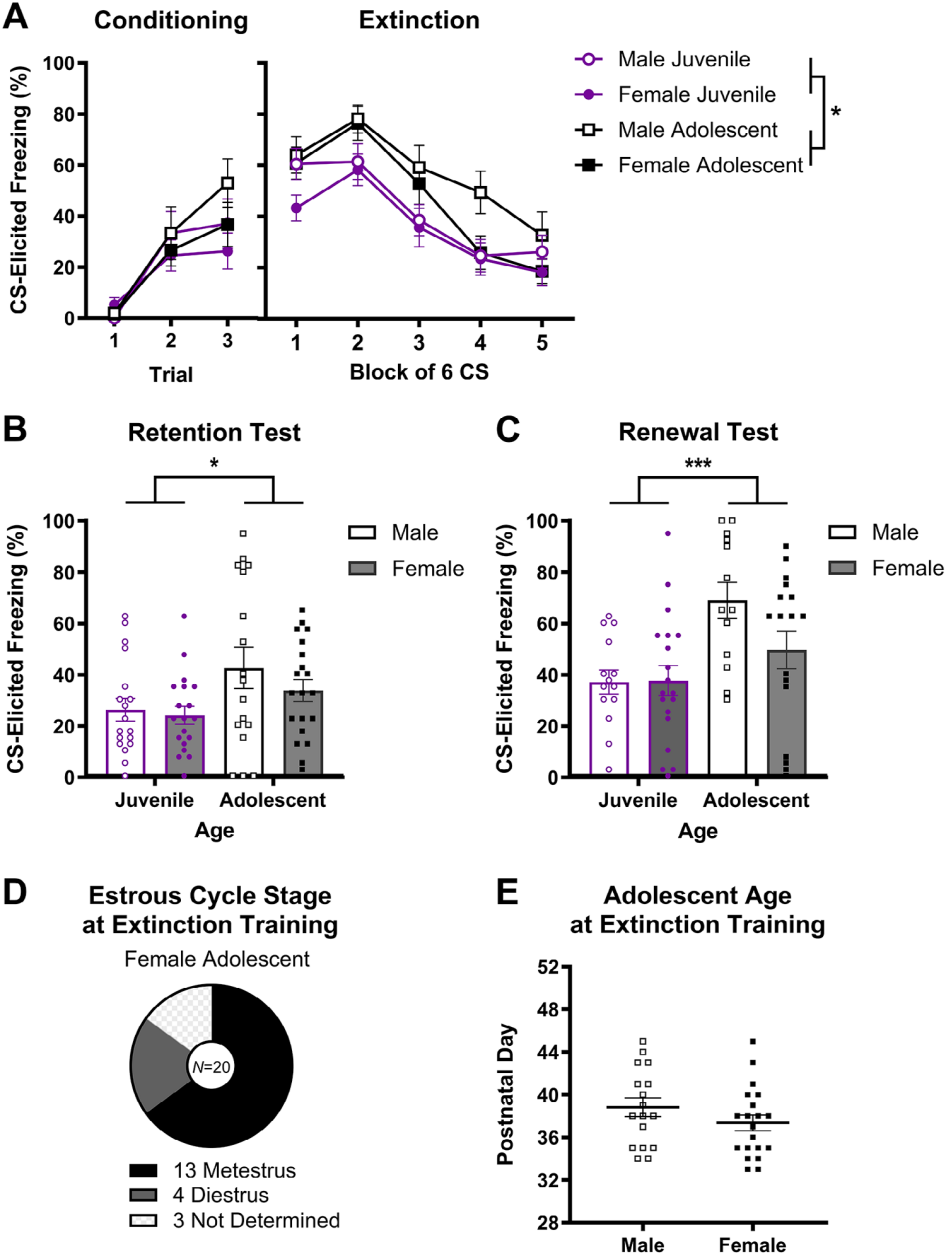
Fear extinction and renewal in juvenile and adolescent rats. (A) CS‐elicited freezing across conditioning and extinction training. Adolescents had higher levels of CS‐elicited freezing across extinction training than juveniles, regardless of sex, but a similar rate of within‐session extinction. (B and C) CS‐elicited freezing at the retention and renewal tests. Adolescents had higher freezing than juveniles when extinction retention and renewal were tested. (D) The number and proportion of adolescent females in each estrous stage at extinction training, with most being metestrus. (E) Male and female adolescents received extinction training at an equivalent age. Group sizes for conditioning, extinction training, and the retention test were *n* = 18 for male juveniles, *n* = 19 for female juveniles, *n* = 17 for male adolescents, and *n* = 20 for female adolescents (except for the renewal test, where some group sizes were slightly smaller—see caption for Table 2). The data shown are mean ± standard error of the mean. Individual data points are included in Panels B, C, and E. Asterisks indicate a significant main effect of age at *p < 0.05 or ***p = 0.001.

Vaginal cytology indicated that most rats (i.e., 14 out of 20) were in estrus on the day of vaginal opening (i.e., the majority of cells taken via lavage and identified microscopically were cornified cells; see representative images in Figure 2). Five rats were in metestrus, and the stage of one rat could not be determined. The daily assessment of the estrous cycle revealed that most of the female adolescent rats began regular cycling immediately following the onset of puberty and showed a 5‐day cycle of 1 day of estrus, 1 day of metestrus, 2 days of diestrus, and 1 day of proestrus, as is typically seen in female adult rats (Becker et al. 2005). As past work on the estrous stage and fear extinction has reported that the stage at the time of extinction training influences extinction retention (Graham and Milad 2013; Milad et al. 2009), the estrous cycle stage of rats during extinction training in this study was recorded. As shown in Figure 3D, most of the female adolescents in Experiment 1 were in metestrus at extinction training, as indicated by a predominance of leukocytes, often with high cellularity (see Figure 2).

#### 3.1.3 Pre‐CS Freezing

Pre‐CS freezing was low before conditioning, extinction training, and the extinction retention test (all means < 7%) and slightly higher (8%–18%) at the renewal test when animals were tested in the original training context (Table 1). There were no group differences detected in pre‐CS freezing at any time point (largest *F*(1, 70) = 3.33, *p* = 0.07, *η_p_* ^2^ = 0.045, age main effect at conditioning).

**TABLE 1:** Mean (standard error of the mean) pre‐CS freezing levels in Experiment 1.

Session	Male juvenile *n* = 18	Female juvenile *n* = 19	Male adolescent *n* = 17	Female adolescent *n* = 20
Conditioning	0.42 (0.30)	2.63 (1.44)	0.15 (0.15)	0.13 (0.13)
Extinction training	3.42 (1.03)	6.45 (2.17)	6.76 (2.78)	5.13 (1.90)
Extinction retention	5.42 (1.43)	6.18 (2.06)	6.32 (2.84)	4.63 (1.56)
Renewal	11.79 (3.63)	11.45 (3.67)	17.69 (7.23)	8.38 (2.02)

*Note:* The renewal test had smaller group sizes for three groups: *n* = 14 for the male juvenile group, *n* = 13 for the male adolescent group, and *n* = 17 for the female adolescent group.

#### 3.1.4 Conditioning

The results for fear conditioning are displayed in Figure 3A. Freezing to the CS increased across conditioning, as confirmed by a significant main effect of conditioning trial in a mixed model ANOVA (*F*(2, 140) = 44.74, *p *< 0.001, *η_p_* ^2^ = 0.390). All groups had a comparable level of overall freezing across conditioning and a similar rate of acquisition, which was confirmed by the nonsignificant main effects for age (*F* < 1) and sex (*F*(1, 70) = 1.49, *p =* 0.23, *η_p_* ^2^ = 0.021), as well as nonsignificant interactions (largest *F*(2, 140) = 2.08, *p* = 0.129, *η_p_* ^2^ = 0.029, sex by trial interaction).

#### 3.1.5 Extinction Training

Figure 3A depicts the data from extinction training. All groups displayed a gradual decrease in CS‐elicited freezing across extinction blocks, but adolescents, overall, had higher levels of freezing during extinction training relative to juveniles. This description of the data was confirmed by a mixed‐model ANOVA. There was a significant main effect of extinction block (*F*(3.22, 225.23) = 51.01, *p *< 0.001, *η_p_* ^2^ = 0.422), indicating that CS‐elicited freezing declined across extinction training across groups. There was also a main effect of age (*F*(1, 70) = 6.69, *p* = 0.012) of a medium effect size (*η_p_* ^2^ = 0.087), with adolescents having higher overall CS‐elicited freezing than juveniles. No main effect of sex or any interactions were detected (largest *F*(1, 70) = 2.74, *p* = 0.102, *η_p_* ^2^ = 0.038, main effect of sex), suggesting a comparable rate of within‐session extinction in males and females. Male and female adolescent rats received extinction training at equivalent ages (*t*(35) = 1.30, p = 0.202, d = 0.429; Figure 3E). In sum, male and female juveniles and adolescents had a comparable rate of extinction, although adolescents overall exhibited higher levels of CS‐elicited freezing during extinction than juveniles.

#### 3.1.6 Extinction Retention Test

The results for the extinction retention test are shown in Figure 3B. As predicted, adolescents had higher CS‐elicited freezing relative to juveniles during the extinction retention test, regardless of sex. This description of the data was confirmed by ANOVA, which detected a main effect of age (*F*(1, 70) = 6.27, *p* = 0.015) of a medium effect size (*η_p_* ^2^ = 0.082), but no main effect of sex (*F*(1, 70) = 1.12, *p* = 0.29, *η_p_* ^2^ = 0.016) or interaction of sex with age (*F* < 1). These findings replicate previous findings that adolescents showed impaired extinction retention relative to juveniles (Baker and Richardson 2015; Kim et al. 2011; McCallum et al. 2010) and extend those studies by demonstrating comparable extinction retention in male and female juvenile rats. This experiment also demonstrated that peri‐pubertal females had a similar deficit of extinction retention as age‐matched males.

#### 3.1.7 Renewal Test

Figure 3C depicts mean CS‐elicited freezing during the renewal test. Adolescents had higher freezing to the CS in the conditioning context relative to juveniles. ANOVA confirmed this description of the renewal data, as there was a main effect of age (*F*(1, 59) = 11.40, *p* = 0.001) of large effect size (*η_p_* ^2^ = 0.162), with adolescents having higher CS‐elicited freezing at the renewal test relative to juveniles. Although peri‐pubertal females appeared to have somewhat lower freezing during the renewal test, ANOVA did not detect any main effect of sex (*F*(1, 59) = 2.08, *p* = 0.16, *η_p_* ^2^ = 0.034) or an age by sex interaction (*F*(1, 59) = 2.36, *p* = 0.13, *η_p_* ^2^ = 0.038), indicating that there were no robust sex differences in fear renewal. Therefore, in this experiment, adolescents had higher CS‐elicited freezing at the renewal test than juveniles, and there were no differences in levels of CS‐elicited freezing in the renewal test in peri‐pubertal and juvenile female rats relative to age‐matched males.

As fear renewal is conceptualized as an increase in fear responses from the extinction context to the original conditioning context, we did a mixed‐model ANOVA with test context as a repeated‐measures factor to compare whether groups differed on the degree of change in CS‐elicited freezing from the extinction retention test to the renewal test. CS‐elicited freezing increased across contexts (*F*(1, 59) = 26.42, *p* < 0.001, *η_p_* ^2^ = 0.309), confirming that renewal was detected across groups. A main effect of age was detected (*F*(1, 59) = 12.89, *p* < 0.001, *η_p_* ^2^ = 0.179), with adolescents having higher overall freezing at tests than juveniles, but no main effect of sex (*F*(1, 59) = 1.09, *p* = 0.30, *η_p_* ^2^ = 0.018), or any interactions were detected (largest *Fs* (1, 59) = 1.61, *p* = 0.21, *η_p_* ^2^ = 0.26, for both the interaction of age and sex as well as the interaction of text context by age). These findings confirm that juveniles and adolescents had fear renewal, and the degree of renewal did not differ by age or sex. In addition, the estrous cycle at renewal did not explain variability in CS‐elicited freezing in adolescent females. Of 17 animals, 10 were in diestrus, 4 were in proestrus, 2 were in estrus, and the stage for one animal could not be determined. High (> 60%) and low levels (< 10%) of freezing at the renewal test were observed in animals in both low (diestrus) and high (proestrus) estradiol stages of the estrous cycle.

### 3.2 Experiment 2

The findings of Experiment 1 indicate that peri‐pubertal female rats have impaired extinction retention, similar to male adolescents, relative to juvenile male and female rats. A likely explanation for the impairment in extinction retention in peri‐pubertal female rats is that most of the animals were extinguished during metestrus, a low estradiol phase of the estrous cycle. Adult female rats extinguished in metestrus have higher levels of CS‐elicited freezing when extinction retention is tested relative to female adults extinguished in proestrus, a high estradiol phase (Graham and Milad 2013; Graham and Scott 2018a; Milad et al. 2009), and so a similar relationship between estrous cycle at extinction training and subsequent extinction retention may occur in adolescent female rats. The aim of Experiment 2 was to examine whether the stage of the estrous cycle at the time of extinction training affects extinction retention in adolescent female rats. It was predicted that adolescent females would exhibit enhanced extinction retention relative to males when extinction occurred in proestrus but not metestrus. Rats were also tested for renewal of extinguished fear. Based on the results of Experiment 1 and previous work demonstrating that female adult virgin rats extinguished in the proestrus and metestrus phases show comparable levels of renewal (Milligan‐Saville and Graham 2016), it was predicted that adolescent rats would exhibit similar levels of CS‐elicited freezing at the renewal test regardless of sex or estrous cycle.

**FIGURE 4:**
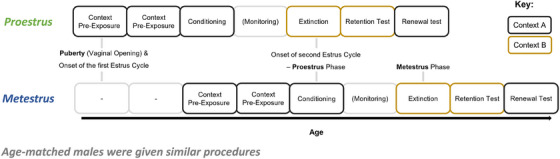
Schedule for the behavioral procedures in Experiment 2. Behavioral sessions occurred on consecutive days except for between conditioning and extinction training, where the estrous cycle was monitored.

A timeline of the experimental procedures is depicted in Figure 4. Female rats were assessed for the onset of puberty, as indicated by vaginal opening, and the estrous cycle was then monitored using similar procedures as in Experiment 1 so that animals received extinction training during either the metestrus or proestrus phase of their second estrus cycle. Given that metestrus typically occurs 2 days later in the estrous cycle than proestrus, the metestrus group started the behavioral procedures 2 days later than the proestrus group. Further, we also allowed an extra day between conditioning and extinction so that extinction training could be timed to occur when rats were in proestrus or metestrus. Rats received tests for extinction retention and renewal on the 2 days after extinction training.

The male adolescent rats were age‐matched to either the female proestrus or metestrus groups, with the males undergoing the timeline of experimental procedures of rats in those groups. Male adolescents were briefly handled each morning in place of the vaginal lavage that was administered to female adolescents.

#### 3.2.1 Exclusions

Three animals (two from the metestrus group and one male) were excluded from the behavioral analyses for low levels of conditioned freezing at the start of extinction (i.e., levels of freezing less than 15% across extinction blocks 1 and 2). One male adolescent and one female from the proestrus group were deemed statistical outliers (i.e., excluded for being more than 2 standard deviations away from the mean during either the extinction retention test or renewal test). Data from the extinction retention test (both pre‐CS and CS‐elicited freezing) of one male rat were missing due to experimenter error. The final group sizes for Experiment 2 were *n* = 18 for the male adolescent and female metestrus group and *n* = 15 for the female proestrus group.

#### 3.2.2 Vaginal Cytology

Vaginal opening occurred between P31 and P43 (*M* = 37.73, *SD* = 3.86) for rats in the female proestrus group and between P31 and P41 (*M* = 36.22, *SD* = 3.73) for the metestrus group. The age at the onset of puberty did not differ across groups (*t*(31) = 1.14, p = 0.263, *d* = 0.40). Daily assessment of the estrous cycle indicated that female adolescents in both groups had regular cycling immediately after the onset of puberty. Replicating findings in Experiment 1, estrus was the predominant phase on the day of vaginal opening (i.e., observed in 22 out of 33 rats; 11 in metestrus), and thereafter, rats typically cycled through the stages of metestrus, 2 days of diestrus, and then proestrus.

#### 3.2.3 Pre‐CS Freezing

Levels of pre‐CS freezing were generally low during conditioning, extinction training, and the extinction retention test. As in Experiment 1, levels of pre‐CS freezing were, not surprisingly, highest before the renewal test (Table 2). There were no significant differences between groups in levels of pre‐CS freezing at any time point in the experiment (largest *F*(2, 47) = 2.43, *p* = 0.10, *η_p_* ^2^ = 0.094, group effect at extinction retention).

**TABLE 2:** Mean (standard error of the mean) pre‐CS freezing levels in Experiment 2.

Stage	Male adolescents (*n* = 18)	Female metestrus (*n* = 18)	Female proestrus (*n* = 15)
Conditioning	0 (0)	0.14 (0.14)	0 (0)
Extinction training	2.36 (0.96)	4.17 (1.94)	1.50 (0.84)
Extinction retention	7.35 (2.25)	7.22 (2.89)	1.00 (0.59)
Renewal	19.17 (5.66)	13.06 (5.56)	5.33 (1.71)

#### 3.2.4 Conditioning

**FIGURE 5:**
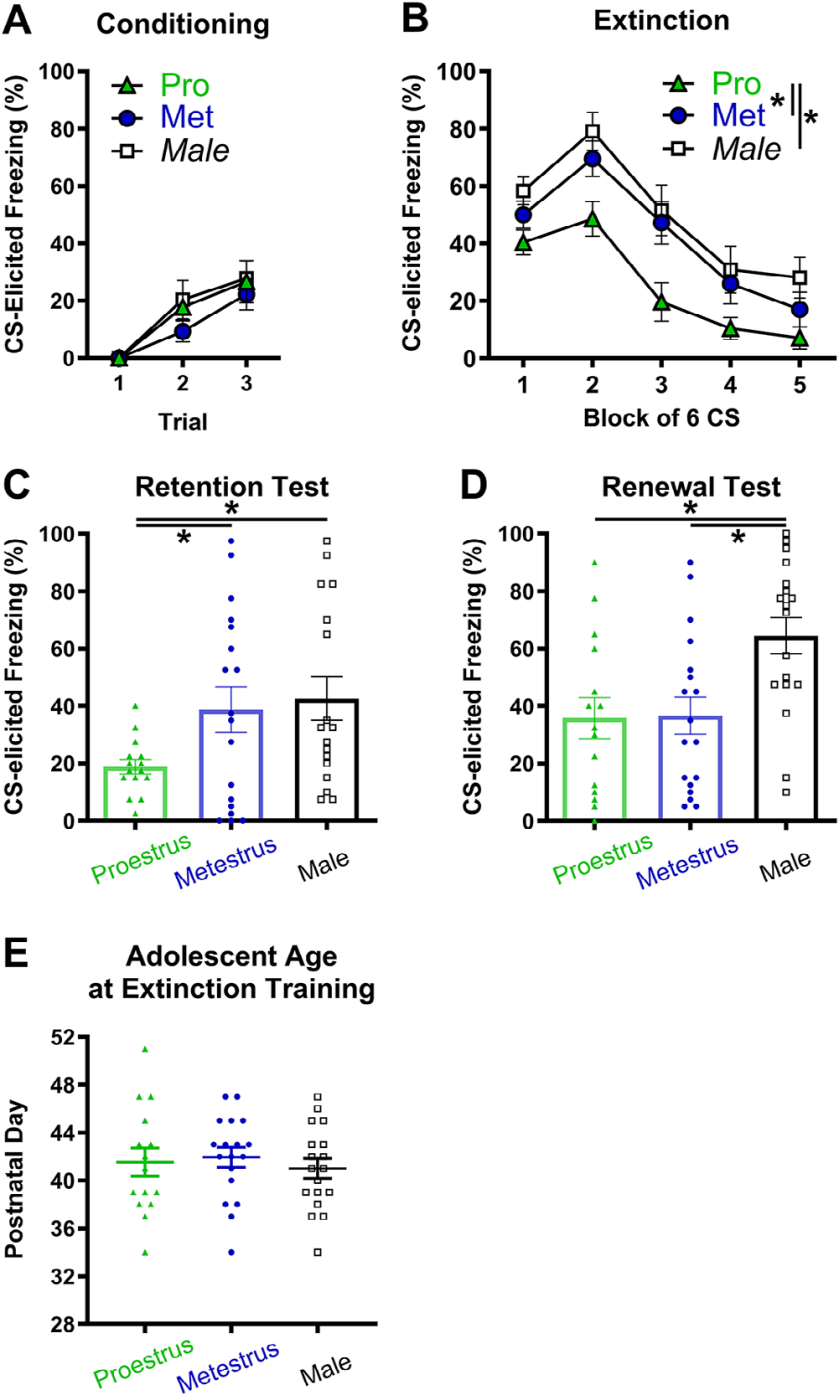
Fear extinction and renewal in female adolescent rats were extinguished in proestrus (Pro) or metestrus (Met) relative to age‐matched males. (A and B) CS‐elicited freezing across conditioning and extinction training. Females receiving extinction training in proestrus had lower freezing during extinction training than those in metestrus and males. (C) Female adolescents receiving extinction in proestrus had lower CS‐elicited freezing than males and metestrus females when extinction retention was tested. (D) Females receiving extinction during either proestrus or metestrus had lower CS‐elicited freezing than males at the renewal test. (E) All groups of adolescents received extinction training at an equivalent mean age. Group sizes were *n* = 18 for males (except the extinction retention test, where *n* = 17), *n* = 18 for metestrus females, and *n* = 15 for proestrus females. The data shown are mean ± standard error of the mean. Individual data points are also shown in Panels C, D, and E. * *p *< 0.05.

The data from the conditioning session in Experiment 2 are shown in Figure 5A. All groups displayed a gradual increase in CS‐elicited freezing across conditioning trials. A mixed‐model ANOVA confirmed this description of the data, as there was a significant main effect of conditioning trial (*F*(2, 96) = 26.34, *p *< 0.001, *η_p_* ^2^ = 0.354). The analysis did not reveal a main effect of group (*F* < 1) or an interaction of group and trial (*F* < 1), suggesting that overall freezing between groups was similar and that all groups had comparable rates of conditioning.

#### 3.2.5 Extinction Training

Figure 5B illustrates the mean CS‐elicited freezing for extinction training in Experiment 2. Females in proestrus appeared to have the lowest levels of freezing across extinction blocks, in comparison to females extinguished in metestrus and males. An ANOVA confirmed an effect of group (*F*(2, 48) = 6.09, *p* = 0.004) with a large effect size (*η_p_* ^2^ = 0.202). Post hoc tests, with the SNK procedure, revealed that females extinguished in proestrus had lower average levels of CS‐elicited freezing during extinction training than both females extinguished in metestrus and male adolescents (*p* < 0.05). Overall levels of CS‐elicited freezing were comparable in the groups of male adolescents and females in metestrus (*p* = 0.278). All groups displayed a decrease in CS‐elicited freezing across extinction blocks (main effect of block: *F*(4, 192) = 53.14, *p *< 0.001, *η_p_* ^2^ = 0.525) and a comparable rate of extinction (block × group interaction: *F* < 1). These results suggest CS‐elicited freezing during extinction was reduced in females relative to males when females received extinction in a high estradiol phase but not a low estradiol phase. These effects were unrelated to age, as the average age of rats at the time of extinction training did not differ significantly across groups (*F* < 1; Figure 5E).

#### 3.2.6 Extinction Retention Test

There were group differences at the extinction retention test (Figure 5C), with female rats showing the lowest levels of CS‐elicited freezing when extinguished in proestrus and males exhibiting the highest levels of freezing. A one‐way ANOVA detected a significant effect of group (*F*(2, 47) = 3.29, *p* = 0.046) of a medium effect size (*η_p_* ^2^ = 0.123). Post hoc tests revealed that females extinguished in proestrus had lower average levels of CS‐elicited freezing at the retention test than both females extinguished in metestrus and male adolescents (*p* < 0.05). CS‐elicited freezing was comparable in females in metestrus and male adolescents (*p* = 0.67). These results suggest that female adolescents have enhanced extinction retention relative to adolescent males when extinguished in proestrus, but not metestrus.

#### 3.2.7 Renewal Test

A different pattern of results emerged for mean CS‐elicited freezing at the renewal test compared to the extinction retention test. Contrary to predictions, male adolescents exhibited strikingly higher levels of freezing to the CS presented in the conditioning context after extinction relative to female adolescents, regardless of the stage of the estrous cycle at extinction training. There was a significant effect of group (*F*(2, 48) = 6.28, *p* = 0.004) of a large effect size (*η_p_* ^2^ = 0.207). Post hoc tests confirmed that male adolescents had higher levels of CS‐elicited freezing at the renewal test than both female metestrus adolescents and female proestrus adolescents (*p* < 0.05), and that the two female groups did not differ (*p* = 0.92). These results suggest that female adolescents extinguished in their second estrous cycle, regardless of stage, have lower levels of CS‐elicited freezing at a renewal test than age‐matched males.

A mixed‐model ANOVA with test context as a repeated‐measures factor was conducted to compare whether groups differed in the degree of change in CS‐elicited freezing from the extinction retention to the renewal test. CS‐elicited freezing increased across contexts (*F*(1, 47) = 11.62, *p* = 0.001, *η_p_* ^2^ = 0.198), confirming that renewal was detected across groups. A main effect of group (*F*(2, 47) = 5.80, *p* = 0.006, *η_p_* ^2^ = 0.198) and interaction of group by test context were also detected (*F*(2, 47) = 4.33, *p* = 0.019, *η_p_* ^2^ = 0.155). Follow‐up simple main effects identified the source of the interaction. Higher levels of freezing were observed in Context A than in Context B, indicative of renewal, for rats extinguished during proestrus (*p* = 0.021) and males (*p* < 0.001). In contrast, rats extinguished during metestrus (*p* = 0.78) had comparable levels of freezing in the two contexts. These findings suggest that although proestrus females had low levels of freezing in both tests, they did have some renewal of extinguished fear.

#### 3.2.8 Associations of Age at Puberty and Extinction Training With Extinction Retention and Renewal

**FIGURE 6:**
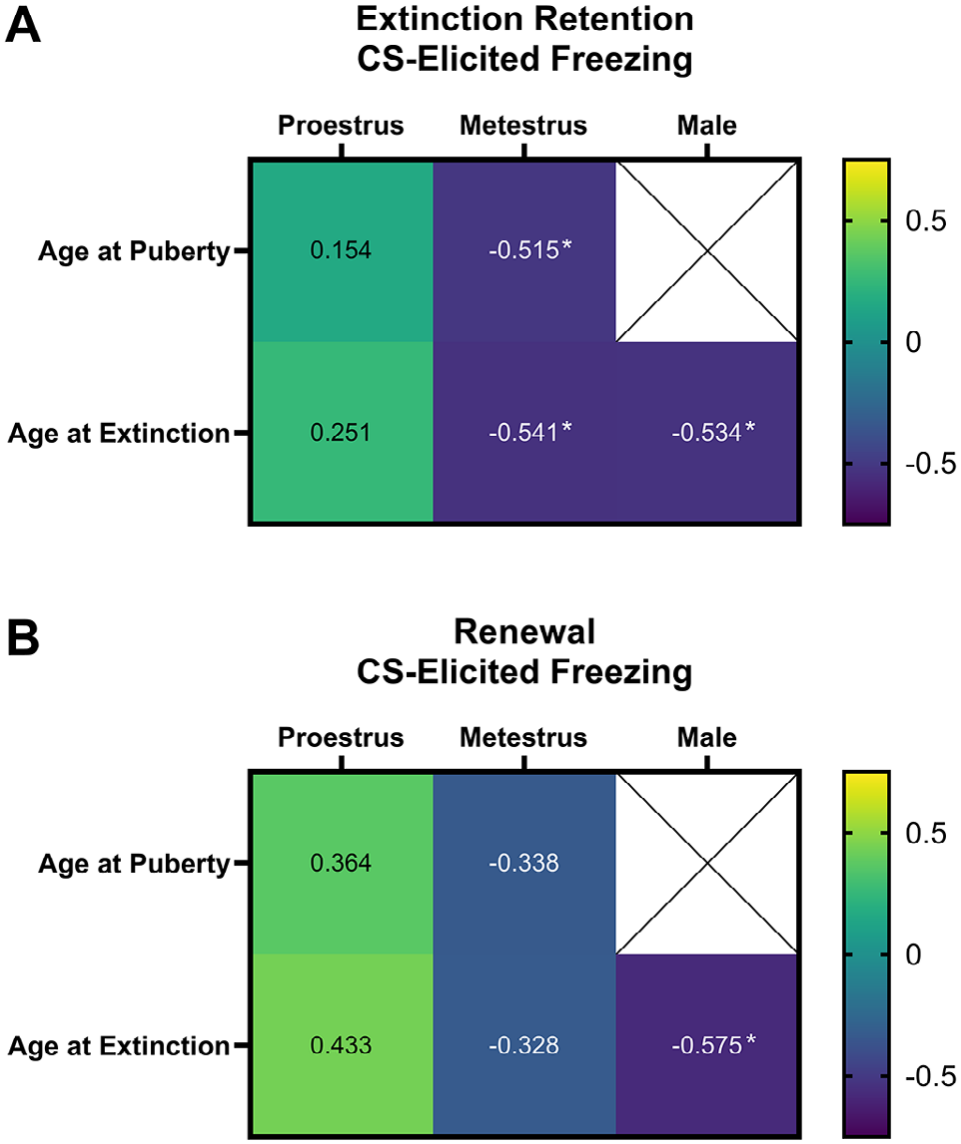
Heatmap of correlations of age at puberty and extinction training with extinction retention (A) and renewal (B) in adolescents. Female rats received extinction training in either proestrus or metestrus. An asterisk indicates Spearman's correlation *r* values are *p* < 0.05.

Figure 6 illustrates the associations between age at puberty and extinction training with freezing at the two tests for adolescent males and females. CS‐elicited freezing at extinction retention was negatively correlated with age at puberty (*r*_16_ = −0.515, *p* = 0.029, 95% CI [−0.784, −0.150]) and extinction training (*r*_16_ = −0.541, *p* = 0.020, 95% CI [−0.840, −0.104]) in metestrus females. In contrast, CS‐elicited freezing at the extinction retention test did not correlate with age at puberty (*r*_13_ = 0.154, *p* = 0.59; 95% CI [−0.367, 0.693]) or age of extinction training (*r*_13_ = 0.251, *p* = 0.37; 95% CI [−0.270, 0.724]) in proestrus females. Fear at the renewal test was not associated with either age at puberty or extinction in either proestrus or metestrus females (largest *r*_13_ = 0.433, *p* = 0.11; 95% CI [−0.378, 0.803]). These findings suggest that older female adolescents undergoing extinction during metestrus are more likely to have successful long‐term extinction retention than female adolescents undergoing extinction when younger. Earlier pubertal onset was associated with poorer extinction retention when extinction occurred in metestrus but not proestrus.

In males, age at extinction training was negatively correlated with freezing at extinction retention (*r*_15_ = −0.534, *p* = 0.027, 95% CI [−0.874, 0.029]) and renewal (*r*_15_ = −0.575, *p* = 0.016, 95% CI [−0.836, −0.273]), indicating that older male adolescents were more likely to have successful long‐term extinction and less context‐mediated relapse than younger male adolescents.

### 3.3 Experiment 3

Experiment 2 demonstrated that female adolescent rats have enhanced extinction retention compared to male adolescent rats when extinguished in proestrus but not metestrus. The latter finding is consistent with the observation in Experiment 1 that most female animals that received extinction the day after pubertal onset were in metestrus and exhibited impaired extinction retention, comparable to same‐age males, the following day. A likely explanation for these results across experiments is that the proestrus females were extinguished during a phase that in adult females, has higher estradiol than the metestrus phase, and these hormonal levels are associated with improved consolidation of extinction. The aim of Experiment 3 was to investigate whether serum estradiol levels in adolescent females were indeed higher during the proestrus phase relative to metestrus of their second cycle and the first 2 days after pubertal onset (i.e., on the day of vaginal opening and the day following vaginal opening). We predicted that adolescent females would have low levels of estradiol on the day following vaginal opening, a timepoint corresponding to when extinction training was given in Experiment 1, but would be higher when the vaginal cytology indicated the animal was in estrus.

Pubertal onset and the estrous cycle were monitored in adolescent females using the previously established procedures. Animals were euthanized on either the day of vaginal opening, the day after vaginal opening, the day of proestrus during the second cycle, or the day of metestrus during the second cycle. Estradiol levels in serum were measured using ELISA. The group sizes were *n* = 13 for the Day of Vaginal Opening group, *n* = 15 for the Day after Vaginal Opening group, *n* = 19 for the Proestrus group, and *n* = 15 for the Metestrus group.

**FIGURE 7:**
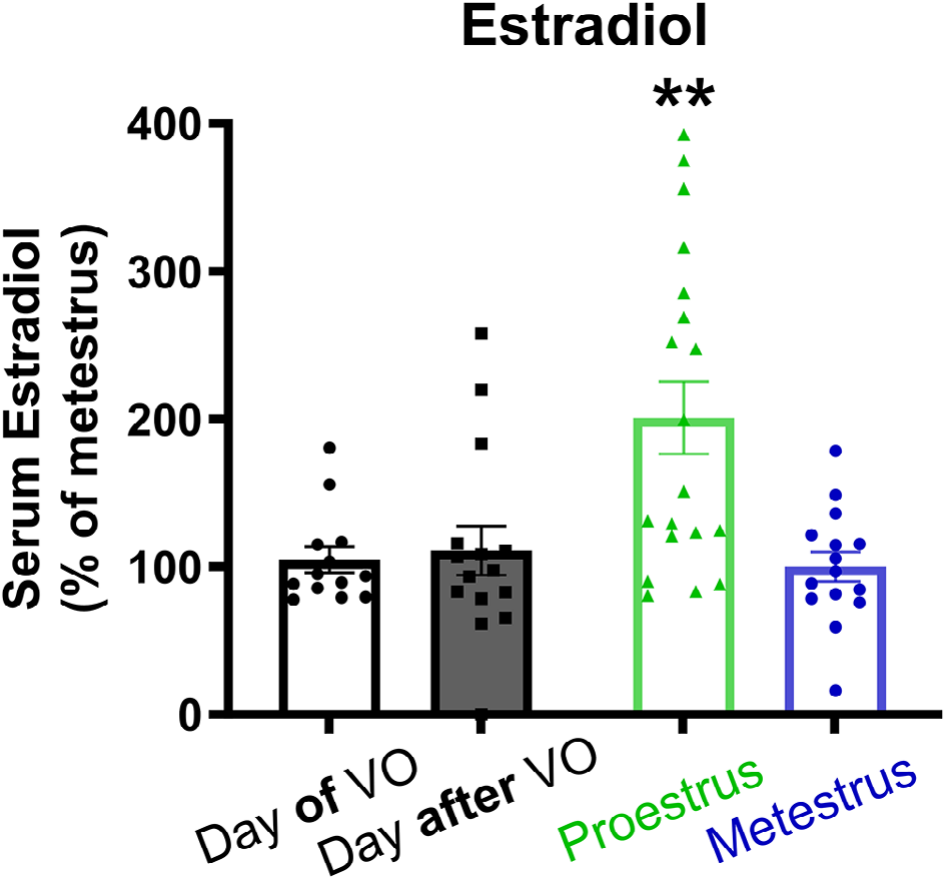
Serum estradiol levels are elevated in female adolescent rats during the proestrus stage of the estrous cycle relative to metestrus and on the day of and the day after vaginal opening (VO). The bars represent the mean (± standard error of the mean). Individual data points are also shown. Group sizes were *n* = 13 for Day of VO, *n* = 15 for Day after VO, *n* = 19 for Proestrus, and *n* = 15 for Metestrus. **indicates *p *< 0.01 compared to all other groups.

Figure 7 illustrates that serum estradiol levels were elevated in proestrus relative to metestrus as well as on the day of and the day after vaginal opening. A one‐way ANOVA confirmed that estradiol levels differed across groups (*F*(3, 58) = 7.89, *p* < 0.001) of a large effect size (*η_p_* ^2^ = 0.290). Tukey's HSD *post hoc* tests indicated that serum estradiol levels were higher in proestrus relative to metestrus (*p* = 0.001), on the day of vaginal opening (*p* = 0.002), and the day after vaginal opening (*p* = 0.003). Estradiol levels were equivalent between all groups other than proestrus (all *p *≥ 0.975). These findings confirm that serum estradiol levels in adolescent females are higher in proestrus than in metestrus and on the first 2 days after pubertal onset. These results support the conclusion that differences in extinction retention across the estrous cycle and at pubertal onset in female adolescents observed in the previous experiments are likely related to fluctuations in circulating estradiol levels.

## 4 Discussion

In this study, we found that female rats were protected against impairments in extinction retention in adolescence when extinction training occurred during periods of high circulating estradiol. Before puberty, female juvenile rats had similar extinction retention as male juveniles. We replicated previous findings that male adolescent rats have an extinction retention deficit relative to juveniles (Baker and Richardson 2015; Kim et al. 2011; McCallum et al. 2010). In contrast to those previous reports where no age differences in freezing during extinction training were detected, in this study, adolescent male and female rats had higher levels of freezing during extinction training compared to juveniles, like what has been reported in adolescent mice relative to younger and/or older animals (Hefner and Holmes 2007; Pattwell et al. 2012). We also found that peri‐pubertal female rats had similar levels of fear at the renewal test as male adolescents, and adolescents overall had higher fear at the renewal test than juveniles. When extinction was timed to occur in specific estrous cycle stages, female adolescents extinguished in proestrus had lower levels of CS‐elicited freezing during extinction training and extinction retention compared to females extinguished in metestrus and age‐matched males; however, both groups of females had lower levels of CS‐elicited freezing during the renewal test than males. Finally, serum estradiol levels in adolescent females were confirmed to be elevated in proestrus compared to either metestrus or the first 2 days after pubertal onset. Our findings indicate that female adolescent rats have reduced fear responses both during extinction training and retention relative to males only when extinguished in a high‐estradiol phase. Taken together, these findings suggest fear inhibition may fluctuate across the estrous cycle in adolescence and that estradiol may have a protective effect in females against impairments in fear extinction retention during this developmental period.

In our first experiment, we found developmental differences in extinction and fear at extinction retention and renewal tests, but not acquisition of fear, in early adolescent rats compared to juveniles. In contrast, no sex differences were detected. The similar acquisition of conditioned fear in juveniles and adolescents aligns with previous reports in male rats (Baker and Richardson 2015; Bisby et al. 2018; Kim et al. 2011; McCallum et al. 2010) and mice (Pattwell et al. 2012). Those studies in rats concluded that adolescents exhibit impairments in the consolidation of extinction, as impairments in extinction retention were detected in the absence of age differences in fear expression or extinction learning. In contrast, our study found higher CS‐elicited freezing during extinction training but a similar rate of within‐session extinction in adolescent male and female rats compared to juveniles, like what has been reported in adolescent mice relative to younger and/or older animals (Hefner and Holmes 2007; Pattwell et al. 2012). The larger sample size in this study (*n *= 37 collapsed across sex) may have enabled the detection of a medium‐sized age effect (*η_p_* ^2^ = 0.087), which was a statistically nonsignificant trend in two of our previous studies (*n* = 23–24 in Bisby et al. 2020; and *n* = 5–7 but a very large effect size *η_p_* ^2^ = 0.311 in Zimmermann et al. 2023). We cannot conclusively state that there were age differences in extinction learning/retention in the present study, given the developmental differences in CS‐elicited freezing across extinction training and the lack of differences in the rate of extinction. It is possible that the observed differences reflect developmental differences in fear expression rather than learning and consolidating extinction. Rodents exhibit various behavioral responses to aversive cues, including darting, conditioned suppression, and ultrasonic vocalization (McDannald 2023). Therefore, juveniles could express different behavioral fear responses than adolescents. Although we only measured freezing, in previous work, we observed that the active defensive response of darting was rare in juvenile, adolescent, and adult male rats during extinction training (Zimmermann et al. 2023). In contrast, developmental differences were observed in the exploratory behavior of rearing, as juveniles reared more often than adolescents and adults during extinction training, but adults reared more often than younger ages during the baseline period (Zimmermann et al. 2023). However, given that many past studies have reported differences in extinction retention in adolescents relative to younger and/or older age groups, with no developmental differences in initial expression of the learned fear response (Baker and Richardson 2015; Kim et al. 2011; McCallum et al. 2010), it is possible, and even perhaps likely, that adolescent impairments in learning/consolidating extinction contributed to our results.

Examination of the vaginal cytology indicated that most of the females that received extinction the day after pubertal onset (as assessed by vaginal opening) were in the metestrus phase of the estrous cycle. Therefore, the impaired extinction retention seen in female adolescents, like that of males, may be due to relatively low circulating estradiol levels. In a separate cohort, we confirmed that serum estradiol was lower on the first 2 days after pubertal onset (corresponding to when conditioning and extinction training occurred in Experiment 1) than in the next proestrus stage, suggesting that low levels of estradiol at extinction training may be a likely contributing factor for why extinction retention was impaired in peri‐pubertal females. Nevertheless, estradiol levels do not fully explain this effect, given that prepubertal female juveniles likely also had low levels of estradiol. Estradiol levels double at puberty relative to prepubertal levels in P28 rats transitioning out of juvenility (Parker and Mahesh 1976). The relatively good extinction retention in juveniles relative to peri‐pubertal females must be driven by other neurobiological factors. One possibility is developmental differences in the functional connectivity of the medial prefrontal cortex and amygdala. Juvenile rodents exhibit greater recruitment and plasticity in the infralimbic cortex with fear extinction compared to adolescents (Baker and Richardson 2015; Kim et al. 2011; Pattwell et al. 2012), and it has been proposed that they engage a “fear release/pro‐exploratory” projection from Layer 5 infralimbic cortex to the central amygdala more than do older age groups (Chen et al. 2021; Zimmermann et al. 2023).

A novel finding of this study is that adolescents exhibited more freezing at the renewal test than juveniles, with no differences between sexes, but both ages exhibited a similar degree of renewal of fear from the extinction to conditioning context. The age effect contrasts with one previous report where male juvenile, adolescent, and adult rats exhibited similar levels of freezing at a renewal test, but in that study, no age differences in extinction learning were detected (Kim et al. 2011). Although the extinction and test procedures were relatively similar to the study in males (Kim et al. 2011) and another demonstrating renewal in female juveniles (Park et al. 2020), in the current study, we detected higher overall levels of fear during extinction training in adolescents than in juveniles, even though a slightly weaker strength footshock US was used. Therefore, the age differences in fear at the renewal test may relate to performance during extinction training, the weaker conditioning procedure, and/or a larger sample. Although there is a paucity of research on the renewal of fear in children and adolescents, as noted by others (Treanor et al. 2021), a related line of research demonstrates that the ability to discriminate between threat and safety cues increases after age 10. That is, children younger than 10 have difficulty discriminating between cues signaling danger (CS+) versus safety (CS−) (Glenn et al. 2012; Jovanovic et al. 2014; Waters et al. 2017). Changes in functional connectivity between the prefrontal cortex and amygdala around age 10 (Gee et al. 2013) and in rats from juvenility to adolescence (Zimmermann et al. 2023) may be related to the maturational changes in generalizing fear across stimuli and contexts.

Our second experiment demonstrated that the estrous cycle affects fear expression during extinction training and extinction retention in mid‐adolescent female rats. Specifically, females extinguished in the proestrus phase had enhanced extinction retention compared to both females extinguished in metestrus and males. This result may relate to the lower CS‐elicited freezing during extinction training in proestrus females relative to metestrus females and males. Adult females extinguished in proestrus occasionally have lower levels of CS‐elicited freezing during extinction training than animals extinguished in other phases of the estrous cycle (Gruene et al. 2015; Milad et al. 2009), an effect sometimes related to differences in conditioning (Milad et al. 2009). However, in several other studies, no effect of the estrous cycle has been detected on CS‐elicited freezing levels during extinction training (Graham and Daher 2016; Graham and Scott 2018b). In any case, our findings contrast with a previous report in adolescent rats (P36 at extinction training) that relative to males, proestrus and metestrus/diestrus females had higher CS‐elicited freezing during extinction training, and metestrus females had poorer extinction retention (Perry et al. 2020). In contrast, females in estrus had equivalent extinction learning and retention as males (Perry et al. 2020). Estrous cycle length and stages can be highly variable in early adolescence (Perry et al. 2020), and some of the animals classified as being in estrus in that study had skipped proestrus and could have been transitioning into estrus with elevated estradiol levels. Also, unlike that study, we did not group females in metestrus with those in diestrus, and so females in our study with rising estradiol levels transitioning from diestrus to proestrus were more likely to be allocated to proestrus than to be grouped with those in metestrus. Our adolescents were also on average around 6 days older (∼P42 at extinction training) and may have had more adult‐like stable cycles. These variations may have contributed to the difference in the stage at which optimal extinction in female adolescents was detected. More work examining the influence of the estrous cycle on fear expression and fear extinction during adolescence may yield further insight.

The enhanced within‐session extinction in proestrus females is speculated to at least partly relate to the anxiolytic effects of ovarian hormones. Meta‐analysis confirms a robust effect of the estrous cycle on anxiety‐like behavior, with female rats and mice tested in metestrus/diestrus (lower ovarian hormones) having more anxiety‐like behavior than those in proestrus (higher ovarian hormones) (Pestana and Graham 2024). Experimental manipulations demonstrating that estrogen receptor B agonists accelerate within‐session extinction (Chang et al. 2009) and that exogenous estradiol reduces anxiety‐like behavior (Walf and Frye 2006) in ovariectomized females with chronically low estradiol and progesterone lead to similar conclusions. Similar anxiolytic effects of ovarian hormones may exist in female adolescents.

We replicated in mid‐adolescents the robust enhancement of extinction retention seen when extinction occurs in proestrus relative to low hormonal phases like metestrus reported in adult nulliparous female rats (Graham and Milad 2013; Graham and Scott 2018a; Milad et al. 2009; Milligan‐Saville and Graham 2016; Rey et al. 2014) and also relative to males (Chang et al. 2009). As noted above, these effects differ, for unknown reasons, from the estrous cycle effects in early adolescents reported by Perry et al. (2020). In naturally cycling adult females, cyclical increases in estradiol appear to facilitate fear extinction, whereas cyclical increases in progesterone appear to induce fear extinction impairments (Graham and Daher 2016). Future work may establish whether exogenous estradiol administered before extinction training enhances extinction retention in female adolescents extinguished in metestrus, which has been reported in nulliparous adult females (Graham and Daher 2016; Milad et al. 2009). Estradiol's augmentation of extinction appears related to activation of NMDA receptors, which are typically necessary for extinction consolidation, but not always (e.g., in reproductively experienced female rats, Tang and Graham 2019). NMDA receptors and activation of the infralimbic prefrontal cortex are both associated with improvements in extinction retention following extended extinction training in male adolescent rats (Baker and Richardson 2017; Kim et al. 2011). Nevertheless, there are some sex differences in the neural mechanisms of fear extinction in adolescents and adults in regions such as the hippocampus, basolateral amygdala, infralimbic cortex, thalamic nucleus reunions, entorhinal cortex, and insular cortex (Glavonic et al. 2023; Gruene et al. 2015; Zhang et al. 2024). For example, extinction in adolescent females does not involve NMDA receptor signaling cascades in the hippocampus, unlike what was found in males (Glavonic et al. 2023). Future work may test whether the improved extinction in proestrus compared to metestrus in female adolescents relates to NMDA receptor activation in regions outside the hippocampus or greater recruitment of the infralimbic prefrontal cortex. Such work may help address the gap in knowledge about how the estrous cycle modulates the neural mechanisms of fear extinction in adolescent females.

A novel and somewhat unexpected finding in this study was that mid‐adolescent females, irrespective of the stage of the second estrous cycle at extinction training, had less freezing in the conditioning context during a fear renewal test compared to age‐matched males, whereas younger juvenile and peri‐pubertal females had levels that did not statistically differ from males during fear renewal tests. The sex differences in older but not younger adolescents may suggest maturational changes or that the effects of ovarian hormones on renewal become more pronounced in the second estrous cycle than in the first. For females extinguished in proestrus, the lower levels of freezing during extinction training and retention likely contributed to this, given that they did exhibit renewal like males. In contrast, females in metestrus did not exhibit any renewal. Our findings in adolescents partially align with reports in adults, where females exhibited less renewal of extinguished conditioned responses than males in aversive (Binette et al. 2022) and appetitive conditioning tasks (Anderson and Petrovich 2015). These sex differences in renewal may relate to the less precise context fear memories in adult females than in males (Keiser et al. 2017) or differences in neural activation of brain circuits relating to context‐dependence of extinction. For instance, females have greater extinction‐induced neural activity in the nucleus reunions (Zhang et al. 2024), and a circuit involving the medial prefrontal cortex to the nucleus reunions to the hippocampus is proposed to suppress the retrieval of hippocampal fear memories. That is, stimulation of the nucleus reuniens reduces renewal, whereas its inactivation promotes relapse of extinguished fear in the extinction context (Totty et al. 2023). The sex differences in neural mechanisms supporting context‐dependence of extinction reported in adults may exist in mid‐adolescents and explain why females of this age exhibit less renewal of extinguished fear than males. However, our findings comparing the degree of renewal in mid‐adolescent females indicate a developmental difference in how the estrous cycle modulates renewal of extinguished fear in mid‐adolescents and adults. Specifically, adult females extinguished in metestrus and diestrus, but not proestrus and estrus, exhibit renewal of fear to a CS in a novel context (Bouchet et al. 2017), but we detected the opposite pattern. The reason for this difference is unknown, but it could relate to levels of estradiol and progesterone being generally lower in young adolescents than in mature female rats (Vetter‐O'Hagen and Spear 2012).

Several recent studies have reported that primiparous adult female rats differ from virgin females in several ways regarding extinction (e.g., the impact of the estrous cycle, the role of NMDA receptors, and the BLA) (Kershaw et al. 2023; Milligan‐Saville and Graham 2016; Tang and Graham 2019). Given that the results reported here show that extinction of learned fear in adolescent females (that have gone through at least one estrous cycle) shares at least one similarity with extinction in primiparous adult females (i.e., reduced renewal of extinguished fear), it will be interesting to determine whether the two groups are similar in other ways (e.g., at behavioral or neural levels).

Our study has certain limitations to consider. Although we detected that the estrous cycle was associated with variations in the effectiveness of extinction in female adolescents and that diminished extinction retention was associated with earlier pubertal onset and younger age when extinction occurred in metestrus, we did not manipulate ovarian hormone levels before (e.g., via gonadectomy as done in other work; Perry et al. 2020) or after puberty to confirm these are underlying mechanisms. In addition, we did not measure ovarian hormone levels in females undergoing extinction to minimize potential disruption to behavior from blood sampling. Nevertheless, this meant that we could not determine if there was a correlation between hormone levels and freezing behavior.

In summary, this study demonstrates that female peri‐pubertal adolescent rats have an extinction retention deficit, relative to juveniles, like males of the same age. Following puberty, fear inhibition fluctuates across the estrous cycle, with extinction facilitated in females during periods high in ovarian hormones and impaired during periods of low ovarian hormones. Mid‐adolescent females also exhibit less freezing in tests of renewal of extinguished fear relative to males. Taken together, these findings suggest that estradiol may have a protective effect in females against impairments in fear extinction retention during adolescence. Our findings in adolescence lend further support to calls to time exposure therapy for anxiety and PTSD to the menstrual cycle to facilitate response to extinction processes (S. H. Li and Graham 2017).

## Conflicts of Interest

Professor Richardson was an Associate Editor of Developmental Psychobiology when this manuscript was first submitted and a coauthor of this article. Dr. Baker is an editorial board member of Developmental Psychobiology and a coauthor of this article. To minimize bias, they were excluded from all editorial decision‐making related to the acceptance of this article for publication. The authors report no other conflicts of interest.

## Data Availability

The data supporting this study's findings are available on reasonable request from the corresponding author.
